# The Royal Army Medical Corps

**Published:** 1920-09-04

**Authors:** 


### THE ROYAL ARMY MEDICAL CORPS.

Before the war there were many who decried the R.A.M.C. as a profession, and considered that it showed a lack of enterprise on the part of the student to suggest it as a possible career. These scoffers maintained that for a man, to whom his profession was more than a means of getting his livelihood, the Service offered no scope for the acquirement and following up of special knowledge, or even for keeping up to date with current medical work. A considerable amount of this misconception was due to sheer ignorance of the facts.

The real truth is that the Service is what the individual likes to make it for himself, and, provided he shows some aptitude and real keenness for specialisation, he has every facility to take up pathological and other research. In fact, for this side of medicine it is harder to find a post which offers so much material and so much variety.

Specialists in surgery are encouraged in every way, and a man wishing to refresh his knowledge in any branch can obtain frequent special leave to take a course in that branch at the large London hospitals.

Outside the Service the specialist has to spend years living on an insufficient pittance before he can attain a position worth having—and even then it is a gamble as to whether he will obtain one or not. In addition, he has often to undertake outside work in his spare time to find the wherewithal to live until his position is assured; whereas the Army specialist has actually an increased allowance made to him on his reaching a certain standard of proficiency.

What is so often forgotten is that the neglect of the individual in taking advantage of his opportunities and succumbing to the temptation to slack and play games is his fault and not that of the Service.

Perhaps for some married men there are serious drawbacks, but for the officer who is one of a large family and who has no ties, the R.A.M.C. has many attractions. A man has to put up no capital to start —a great consideration for the father; he can see the world; he has a very good income without the constant drag of having to save every penny he can, for he has a very handsome pension at the end, varying with his length of service. He has plenty of leave, for which he is paid, whereas the practitioner has to pay for his substitute besides losing much besides.

Furthermore, even if he is so unfortunate as to be stationed abroad where he does not wish to be he can nearly always effect an exchange with a man at home, as the attractions of extra foreign pay are considerable. Finally, if he does not like the Service he can always resign his commission and take up the life of an ordinary practitioner, but with a pension to help him.

